# Probiotic *Lactobacillus reuteri *biofilms produce antimicrobial and anti-inflammatory factors

**DOI:** 10.1186/1471-2180-9-35

**Published:** 2009-02-11

**Authors:** Sara E Jones, James Versalovic

**Affiliations:** 1 Cell and Molecular Biology Program, Baylor College of Medicine, Houston, Texas 77030, USA; 2 Department of Pathology, Baylor College of Medicine, Houston, Texas 77030, USA; 3 Department of Pathology, Texas Children's Hospital, Houston, Texas 77030, USA

## Abstract

**Background:**

Commensal-derived probiotic bacteria inhibit enteric pathogens and regulate host immune responses in the gastrointestinal tract, but studies examining specific functions of beneficial microbes in the context of biofilms have been limited in scope.

**Results:**

*Lactobacillus reuteri *formed biofilms that retained functions potentially advantageous to the host including modulation of cytokine output and the production of the antimicrobial agent, reuterin. Immunomodulatory activities of biofilms were demonstrated by the abilities of specific *L. reuteri *strains to suppress human TNF production by LPS-activated monocytoid cells. Quantification of the antimicrobial glycerol derivative, reuterin, was assessed in order to document the antipathogenic potential of probiotic biofilms. *L. reuteri *biofilms differed in the quantities of reuterin secreted in this physiological state.

**Conclusion:**

*L. reuteri *biofilms secreted factors that confer specific health benefits such as immunomodulation and pathogen inhibition. Future probiotic selection strategies should consider a strain's ability to perform beneficial functions as a biofilm.

## Background

Probiotics are defined by the Food and Agricultural Organization of the United Nations as "live microorganisms which when administered in adequate amounts confer a health benefit on the host" [1,2]." The effectiveness of probiotics is strain-specific, and each strain may contribute to host health through different mechanisms. Probiotics can prevent or inhibit the proliferation of pathogens, suppress production of virulence factors by pathogens, or modulate the immune response. *L. reuteri *is a promising therapy for the amelioration of infantile colic, alleviation of eczema, reduction of episodes of workplace illness, and suppression of *H. pylori *infection [3-9]. *L. reuteri *is considered an indigenous organism of the human gastrointestinal tract and is present on the mucosa of the gastric corpus, gastric antrum, duodenum, and ileum [10,11].

Biofilms or adherent structured microbial communities in the oral cavity and respiratory tract are well-characterized and are associated with respiratory infections, dental caries, and periodontitis [12,13]. In contrast, biofilm-like communities of the gastrointestinal and female urogenital tracts containing beneficial lactobacilli may have a protective role. In bacterial vaginosis, indigenous lactobacilli are replaced with pathogenic biofilms consisting of *Gardnerella vaginalis *and other bacteria [6]. Probiotic *L. reuteri *can displace *G. vaginalis *biofilms and could potentially re-establish protective biofilms in the female urogenital tract [6]. Due to artifactual removal of biofilms by traditional fixatives during specimen processing, studies of gastrointestinal biofilms are sparse. Using non-aqueous fixatives and special techniques, several groups have documented the presence of intestinal biofilms in the mammalian intestine [14-17]. The bacterial composition of biofilm-like communities in the mucus layer or the mucosa-associated microbiota (MAM), differs from the composition of the fecal microbiota, and the human MAM includes lactobacilli [18-20]. Changes in the composition and architecture of the MAM through diet or disease may affect overall health status [20-24]. Denser biofilms were found in patients with inflammatory bowel disease (IBD) when compared to healthy controls, and 60% of the biofilm mass was comprised of the commensal *Bacteroides fragilis *[17]. These studies indicate a need to understand the contributions of individual strains and species to the aggregate function of gastrointestinal biofilms.

This report describes the ability of an established commensal and probiotic organism, *L. reuteri*, to form biofilms *in vitro *and perform potentially beneficial functions as biofilms. Two basic probiotic functions that depend on secreted factors were studied in the context of biofilms. First, modulation of innate immunity was investigated by studying regulation of human TNF production. In prior studies, supernatants from planktonic *L. reuteri *cultures reduced production of the pro-inflammatory cytokine, TNF [25], and TNF suppression was important in alleviating inflammation in a murine model of IBD [26]. Probiotic *L. reuteri *biofilms have not been examined for TNF inhibition. Secondly, production of the antimicrobial compound β-hydroxy-propionaldehyde, known as reuterin, was evaluated in order to assess anti-pathogenic properties of *L. reuteri *biofilms.

## Results

### Probiotic *Lactobacillus reuteri *forms biofilms

**Figure 1:**
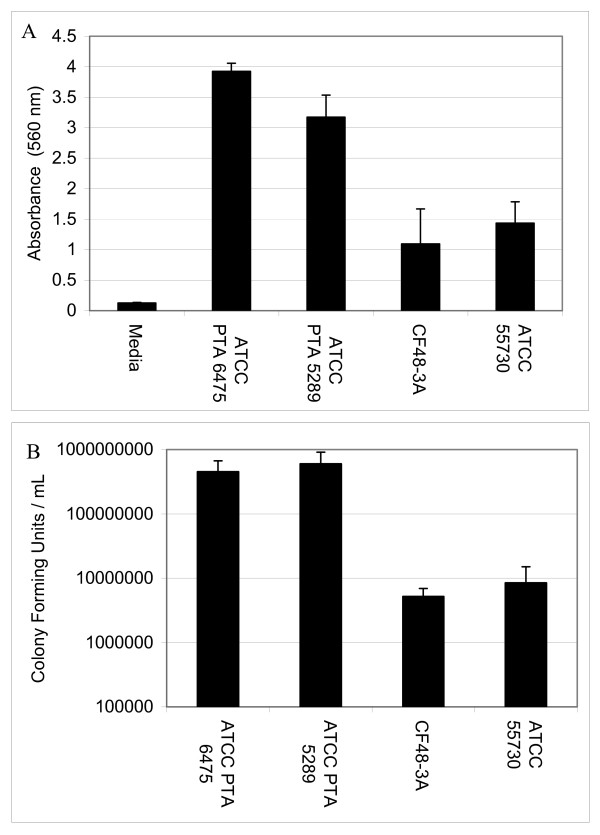
***L. reuteri *adherence is strain-dependent**. *L. reuteri *biofilms were cultured for 24 hours in 96-well polystyrene plates. The relative propensities of *L. reuteri *to form biofilms were measured by absorbance spectrophotometry after staining with crystal violet. *L. reuteri *ATCC 6475 and ATCC PTA 5289 were more adherent then CF48-3A and ATCC 55730 (ANOVA, p < 0.02).

**Figure 2:**
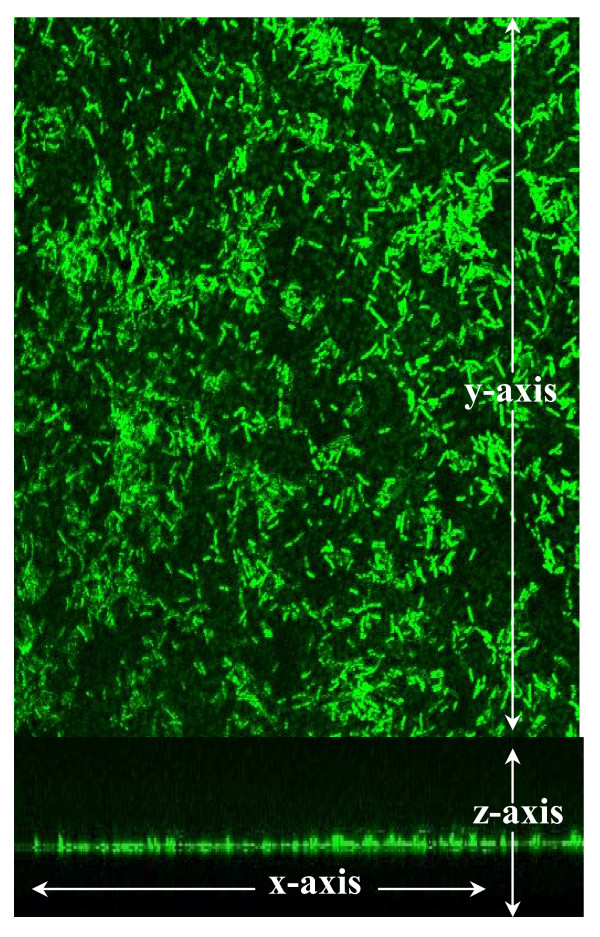
***L. reuteri *biofilms were observed by confocal microscopy**. Biofilms were cultured in a flow cell supplied with MRS for 48 hours at 37°C in ambient atmosphere. *L. reuteri *biofilms (green) were stained with acridine orange and observed by confocal microscopy. A single optical section and the stacked optical sections of ATCC 55730 (**A **and **B**, respectively) are shown at 630× magnification. These images are representative of 30 microscopic fields obtained in 3 independent experiments.

Various human isolates of *L. reuteri *were grown in 96-well polystyrene plates and retention of crystal violet was used to assess relative biofilm densities (Fig. 1A). All strains of *L. reuteri *adhered to polystyrene, but strains differed with respect to relative densities as measured by absorbance spectrophotometry. *L. reuteri *strains ATCC PTA 6475 and ATCC PTA 5289 (OD_570 _was 3.92 and 3.17, respectively) formed aggregates with greater optical densities than *L. reuteri *strains ATCC 55730 and CF48-3A (OD_570 _was 1.10 and 1.44, respectively). The differences between strains were also observed in cell counts. The bacterial densities (CFU/cm^2^) in biofilms of ATCC PTA 6475 and ATCC PTA 5289 were roughly 10-fold greater than the bacterial densities of ATCC 55730 and CF48-3A biofilms (Fig. 1B). *L. reuteri *biofilms were stained with acridine orange and observed by confocal microscopy (Fig. 2). Monospecies biofilms of ATCC 55730 were 7 μm (+/- 2 μm) thick. The thickness of *L. reuteri *biofilms was assessed at 24 and 48 hours. No differences in biofilm thickness were observed. Consistent with this study, other researchers demonstrated formation of only thin biofilms (approximately 5 μm) when *L. reuteri *biofilms were cultured on plastic coupons for 32 hours [27].

### *L. reuteri *biofilms modulate human TNF production

**Figure 3:**
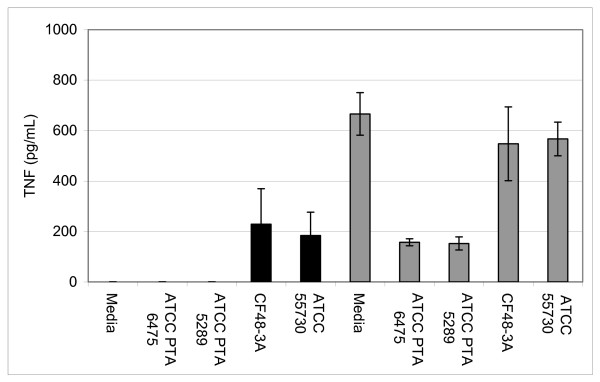
**Modulation of TNF production by *L. reuteri *is strain-dependent**. Cell-free supernatants from stationary phase *L. reuteri *cultures (planktonic cells) were added to human monocytoid cells in the presence or absence of *E. coli*-derived LPS (no LPS-black bars, LPS-gray bars). Quantitative ELISAs measured the amounts of human TNF produced by THP-1 cells. In the absence of LPS, supernatants from *L. reuteri *CF48-3A and ATCC 55730 stimulated TNF by human THP-1 cells, while supernatants from ATCC PTA 6475 and ATCC PTA 5289 did not induce TNF production. However, *L. reuteri *CF48-3A and ATCC 55730 did not suppress TNF production by LPS-activated cells, while PTA 6475 and ATCC PTA 5289 inhibited production of TNF by 76% and 77% respectively, when compared to the media control (ANOVA, p < 0.001).

**Figure 4:**
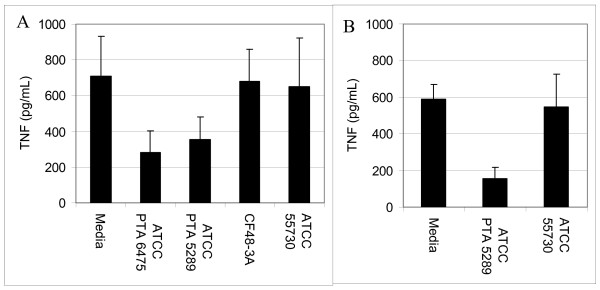
***L. reuteri *strains proficient in biofilm formation suppress TNF production**. Cell-free supernatants from *L. reuteri *biofilms cultured in 24-well plates (**A**) or flow cells (**B**) were added to human monocytoid cells in the presence of *E. coli*-derived LPS. Quantitative ELISAs measured the amounts of human TNF produced by THP-1 cells. As biofilms, TNF inhibitory strains (ATCC PTA 6475 and ATCC PTA 5289) retained their ability to suppress TNF produced by LPS-activated human monocytoid cells. *L. reuteri *ATCC PTA 6475 and ATCC PTA 5289 biofilms cultured in 24-well plates (**A**) inhibited TNF by 60% and 50% respectively, (ANOVA, p < 0.02). Supernatants of *L. reuteri *ATCC PTA 5289 cultured in a flow cell (**B**) inhibited TNF by 73% when compared to the media control (ANOVA, p < 0.0001).

To test the immunomodulatory properties of *L. reuteri *biofilms, supernatants from the biofilms were added to human monocytoid THP-1 cells in the presence and absence of LPS. LPS was added to the THP-1 cells to stimulate production of pro-inflammatory TNF by THP-1 cells. *L. reuteri *strains that produced TNF inhibitory factors as planktonic cultures (*L. reuteri *strains ATCC PTA 6475 and ATCC PTA 5289, 76 and 77%, respectively) (Fig. 3) demonstrated similar abilities to suppress TNF production when cultured as biofilms (Fig. 4). When TNF inhibitory factors were obtained directly from *L. reuteri *biofilms grown in 24-well polystyrene plates, ATCC PTA 6475 and ATCC PTA 5289 also inhibited TNF production by 60% and 50%, respectively, when compared to the media control (Fig. 4A). Supernatants of *L. reuteri *ATCC PTA 5289 biofilms cultured in a flow cell inhibited TNF by 73% compared to the media control (Fig. 4B). *L. reuteri *strains that did not suppress human TNF in planktonic phase (ATCC 55730 and CF48-3A) (Fig. 3) lacked TNF-inhibitory capabilities when supernatants were obtained from the same strains cultured as biofilms (Fig. 4). Surprisingly, supernatants from ATCC 55730 and CF48-3A biofilms did not induce TNF production by THP-1 cells in the absence of LPS (data not shown) as the supernatants from planktonic cultures did (Fig 3). Interestingly, the ability of probiotic *L. reuteri *to regulate human TNF production is strain-specific, and strain-specific TNF inhibition was maintained whether *L. reuteri *strains were cultured as planktonic cells or biofilms. The relative abilities to suppress human TNF in monocytoid cells were directly correlated with relative abilities to aggregate and form biofilms on polystyrene surfaces (Fig. 1A).

### *L. reuteri *cultured as planktonic cells and biofilms produced the antimicrobial factor, reuterin

**Figure 5:**
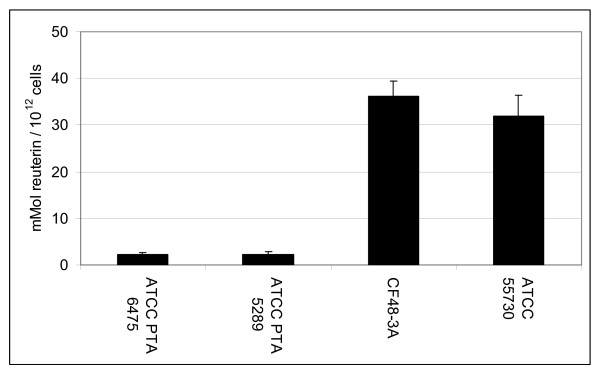
***L. reuteri *strains cultured as planktonic cells produce the antimicrobial compound, reuterin**. Stationary phase planktonic cultures of *L. reuteri *were incubated anaerobically in a glycerol solution. Reuterin concentrations of the cell free supernatants were determined using a colorimetric assay and were normalized with respect to viable colony counts prior to the addition of glycerol. ATCC PTA 6475 and ATCC 5289 produced less reuterin than ATCC 55730 and CF48-3A (ANOVA, p < 0.05).

**Figure 6:**
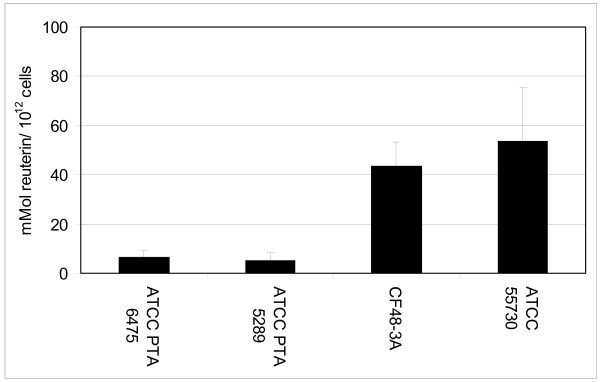
***L. reuteri *biofilms produce reuterin**. *L. reuteri *biofilms were cultured in MRS for 48 hours at 37°C in ambient atmosphere in multiwell plates. In order to measure reuterin production, biofilms were incubated in the presence of glycerol in anaerobic conditions. Reuterin concentrations were determined using a colorimetric assay and were normalized with respect to viable colony counts prior to the addition of glycerol. All *L. reuteri *biofilms produced detectable amounts of reuterin, although inter-strain differences were observed. ATCC PTA 6475 and ATCC 5289 produced less reuterin than ATCC 55730 and CF48-3A (ANOVA, p < 0.05).

Antimicrobial activities of *L. reuteri *were assessed by examining supernatants of planktonic and biofilm cultures for reuterin. Planktonic cells and biofilms of *L. reuteri *produced reuterin, although differences in reuterin production were evident among strains. Planktonic cultures of ATCC PTA 6475, ATCC PTA 5289, ATTC 55730 and CF48-3A produced 51.2, 45.2, 225.9, and 230.3 mM of reuterin, respectively. When reuterin quantities were normalized to initial CFU/mL, planktonic cultures of ATCC PTA 6475 and ATCC PTA 5289 produced 2.32 and 2.3 mmol reuterin/10^12 ^cells, respectively, and ATCC 55730 and CF48-3A produced 31.89 and 36.24 mmol reuterin/10^12 ^cells, respectively (Fig. 5). For biofilms cultured in multiwell plates, the four wild type *L. reuteri *strains ATCC PTA 6475, ATCC PTA 5289, ATTC 55730 and CF48-3A produced 26.8, 16.5, 19.1, and 22.1 mM of reuterin, respectively. After normalization of reuterin quantities to bacterial cell counts, ATCC PTA 6475, ATCC PTA 5289, CF48-3A, and ATCC 55730 produced 6.61, 5.41, 43.4, and 53.94 mmol of reuterin/10^12 ^cells, respectively, when cultured as biofilms in multiwell plates (Fig. 6). Trends in reuterin production were consistent with planktonic and biofilm cultures of ATCC PTA 6475 and ATCC 5289 producing lower quantities of reuterin than strains ATCC 55730 and CF48-3A. Interestingly, the relative abilities of *L. reuteri *strains to produce reuterin were inversely correlated with relative abilities to aggregate and adhere to polystyrene (Fig. 1A).

Previous studies indicated that planktonic cultures of human-derived *L. reuteri *strains used in this study were relatively resistant to the antimicrobial effects of reuterin (10 mM), when compared to other bacterial species including closely related lactobacilli [[29,43] and JK Spinler, unpublished data]. However, since the cell viabilities of planktonic cultures decrease as reuterin accumulates [28], the quantities of reuterin produced by planktonic cultures were normalized to the initial CFU/mL. Reuterin was detected after biofilms were incubated in glycerol for 1, 2, and 3 hours (data not shown). Cell viabilities of biofilms after reuterin production exceeded 92% (data not shown), indicating that the biofilms were relatively resistant to the quantities of reuterin produced by *L. reuteri *biofilms.

## Discussion

Two hallmark features of probiotic function, modulation of cytokine and reuterin production, were examined in this study. Commensal-derived probiotic *L. reuteri *strains formed biofilms, and thesebiofilms retained the probiotic functions observed with planktonic cultures. Single species biofilms composed of anti-inflammatory *L. reuteri *strains ATCC PTA 6475 and ATCC PTA 5289 secreted factors that suppressed TNF production by LPS-activated monocytoid cells. In contrast, biofilms comprised of immunostimulatory probiotic strains ATCC 55730 and CF48-3A lacked the ability to stimulate human TNF production by human cells in the absence of LPS activation. ATCC 55730 and CF48-3A produced greater quantities of reuterin than ATCC PTA 6475 and ATCC PTA 5289 when the bacteria were cultured as planktonic cells or biofilms. Human breast milk-derived strains (ATCC PTA 6475 and ATCC 55730) differed with respect to relative propensities to form biofilms, and these strains demonstrated different biological properties in the context of biofilms.

Lactic acid bacteria secrete factor(s) that inhibit cytokine production by immune cells [26,29-31], and this report established that probiotic biofilms cultured in a variety of conditions produced factor(s) that suppress TNF production by LPS activated human monocytes/macrophages. Previous studies in this lab have demonstrated that the TNF-inhibitory factors are detected in mid-log to stationary phase cultures [32]. Stationary phase cultures yield the most consistent TNF-inhibitory activities (Y.P. Lin, personal communication). Modulation of the mucosal immune system by intestinal commensal bacteria may have important implications for immune homeostasis and biofilm formation [33]. Intestinal bacteria such as *L. reuteri *may stimulate or suppress innate immune responses via several mechanisms including modulation of pro-inflammatory cytokines. *L. reuteri *strains in this study can be divided into two subsets, immunosuppressive (ATCC PTA 6475 and ATCC PTA 5289) and immunostimulatory strains (ATCC 55730 and CF48-3A), and each subset has potential therapeutic value. TNF inhibitory strains of *L. reuteri *reduced inflammation in a *H. hepaticus*-induced murine model of inflammatory bowel disease [26]. By contrast, stimulation of the mucosal innate immune system may be associated with enhanced protection against enteric infections.

Interestingly, mucosal inflammation has been associated with enhanced biofilm densities in the intestine [34,35]. The pro-inflammatory cytokine TNF promotes the proliferation of *E. coli*, and secretory IgA increased agglutination of *E. coli*, an initial step in biofilm development [34,36,37]. Although, these experiments were performed with monospecies biofilms *in vitro*, the data raise questions regarding events that occur in complex microbial communities *in vivo*. When not attached to a surface, immunostimulatory *L. reuteri *strains may stimulate host immune responses and promote commensal biofilm formation, particularly in neonates. When *L. reuteri *biofilms are established, probiotic strains may have a diminished ability to stimulate TNF, effectively suppressing the formation of dense, complex multispecies biofilms in the mucus layer. Because such complex, dense biofilms have been associated with inflammation and disease [17], the ability of probiotics to differentially regulate production of immunomodulatory factors in the context of planktonic and biofilm lifestyles may be an important probiotic feature. Alternatively, the TNF stimulatory factor(s) may be produced by *L. reuteri *biofilms and not detected in the experimental conditions used in this study. In contrast to immunostimulatory *L. reuteri *strains, anti-inflammatory probiotics may form denser biofilms *in vivo *that thwart pathogenic biofilm formation by preventing harmful host:pathogen interactions and overgrowth of commensal bacteria in the intestine. As an example of pathogen inhibition, other lactobacilli suppressed the binding of *Staphylococcus aureus *to epithelial cells [38].

Reuterin is a potent anti-pathogenic compound produced by *L. reuteri *and capable of inhibiting a wide spectrum of microorganisms including gram-positive bacteria, gram-negative bacteria, fungi, and protozoa [39]. Maximum reuterin production by *L. reuteri *occurs during late log and stationary phase cultures (J.K. Spinler, personal communication). Reuterin and other anti-pathogenic factors may be important for maintaining a healthy gut microbiota by preventing intestinal overgrowth by other commensal and pathogenic microorganisms. Recently, the addition of *L. reuteri *ATCC 55730 or reuterin to the intestinal microbiota was shown to reduce the *E. coli *population in an *in vitro *fermentation model [40]. Thus, antimicrobial compounds like reuterin may have a fundamental role in shaping and modeling the composition and spatial architecture of the gastrointestinal microbiota. *L. reuteri *biofilms produced reuterin, indicating that probiotic *L. reuteri *may be protective against pathogens in either the planktonic or biofilm state. Interestingly, strains that produce relatively high quantities of reuterin are immunostimulatory when cultured as planktonic cells. *In vivo*, immunostimulation by *L. reuteri *may promote colonization and biofilm formation of commensal lactobacilli, and reuterin could prevent opportunistic bacteria from establishing a niche. Hypothetically, once the immunostimulatory strains are established on the mucosal surface, TNF stimulation is diminished, and higher quantities of reuterin are produced. Elevated quantities of reuterin adjacent to the mucosa may effectively alter surrounding commensal microbial populations and prevent colonization and adherence by pathogenic bacteria. Biofilms are relatively resistant to several antimicrobial agents when compared to planktonic cultures [41]. The enhanced resistance of biofilms to antimicrobial compounds may explain, in part, the resistance of *L. reuteri *biofilms to reuterin and elevated amounts of reuterin produced by these biofilms, as described in this study.

While the growth conditions used for the flow cell and planktonic cultures differed, similar probiotic activities by each *L. reuteri *strain were observed. TNF inhibitory activities and reuterin production of *L. reuteri *were also consistent when biofilms (in multiwell plates) and planktonic cells were cultured using the same growth conditions. Although these experiments were conducted with biofilms grown *in vitro *on abiotic surfaces, biofilms with probiotic function may be important for delivery of beneficial effects in the mammalian host. A mutant strain of *L. crispatus*, unable to bind mucus and adhere to the colonic mucosa, did not have a protective effect in a murine colitis model compared to the wild type aggregating strain even when the bacteria were continuously supplied to mice [42]. Mucus-binding ability may be important for probiotics to adhere to the mucosal surface and form biofilms within the intestine. Defects in cell surface features may affect biofilm formation and the abilities of probiotics to persist and colonize the intestine *in vivo*. *L. reuteri *strain 100–23 deficient in alanylation of cell surface lipoteichoic acids proliferated similarly to wild type cells as planktonic bacteria, but failed to compete effectively and form complex biofilms *in vivo *[43]. In the context of established intestinal microbial communities, probiotic biofilms may be more effective at long-term colonization and restoring missing functions in disease states.

## Conclusion

In conclusion, probiotic strategies for the prevention and treatment of disease may require discovery and development of strains that form effective biofilms. If biofilm formation facilitates long-term colonization and persistence in the intestine, biofilms that retain probiotic functions may be important for sustained efficacy *in vivo*. The human gastrointestinal microbiota is a complex ecosystem that is shaped and maintained by multiple host and microbial factors. Changes in the spatial distribution, community architecture, or composition of the gastrointestinal microbiota may alter intestinal physiology and immunity, including susceptibility to infection. Probiotics in biofilm-like communities may be essential for long-term remodeling of the composition and function of the intestinal microbiome.

## Methods

### Key reagents, bacterial strains and mammalian cell lines

*L. reuteri *strains were grown in deMan, Rogosa, Sharpe (MRS; Difco, Franklin Lakes, NJ) or LDMIIIG (pH 6.5) (see Additional file 1) media. An anaerobic chamber (1025 Anaerobic System, Forma Scientific, Waltham, MA) supplied with a mixture of 10% CO_2_, 10% H_2_, and 80% N_2 _was used for anaerobic culturing of lactobacilli. Biogaia AB (Raleigh, NC) provided *L. reuteri *strains ATCC PTA 6475, ATCC PTA 5289, ATCC 55730, and CF48-3A. *L. reuteri *ATCC PTA 6475 and ATCC 55730 were isolated from the breast milk of healthy Finnish and Peruvian women, respectively. ATCC PTA 5289 is an oral isolate from a healthy Japanese woman. CF48-3A was isolated from the feces of a healthy Finnish child. THP-1 cells (ATCC TIB-202) were maintained in RPMI 1640 supplemented with 10% fetal bovine serum (Invitrogen, Carlsbad, CA) at 37°C and 5% CO_2_. All chemical reagents were obtained from Sigma-Aldrich (St Louis, MO) unless otherwise stated. Polystyrene 96- and 24-well plates for biofilm and tissue culture studies were obtained from Corning (Corning, NY). Filters with polyvinylidene fluoride membranes (0.22 mm pore size) (Millipore, Bedford, MA) were used for sterilization.

### *L. reuteri *biofilm adherence studies

*L. reuteri *cultured in MRS media for 16–18 hours were diluted 1:50 in MRS to a final volume of 200 μL in sterile 96-well polystyrene plates. Plates were incubated anaerobically at 35°C for 24 hours. Media and planktonic cells were removed by aspiration and two washes with de-ionized water. Adherent cells were stained with crystal violet (0.1% w/v) for 15 minutes at 37°C, 200 rpm. Crystal violet was discarded and the plates were washed with de-ionized water. The crystal violet was redissolved with ethanol and the OD_570 _was determined by absorbance spectrophotometry using a Spectramax 340 PC^384 ^(Molecular Devices, Sunnyvale, CA).

### Confocal imaging of *L. reuteri *biofilms

Glass flow cells with a volume of 7.7 mL (Stovall Inc., Greensboro, NC) were assembled according to the manufacturer's instructions and maintained at 37°C in ambient atmosphere. As previously described, one mL of *L. reuteri *(OD_600 _= 0.1 or 7 × 10^7 ^cells) was injected into the flow cell [44]. *L. reuteri *were allowed to adhere to the glass surface for an hour before being continuously supplied with 25% MRS (v/v) at 2 mL per minute. Cell counts verified that the selected flow rate removed planktonic cells and retained adherent bacteria on the surface of the flow cell. After 48 hours, the flow cells were collected and washed once with sodium phosphate buffer (50 mM) for 10 minutes at 37°C, 70 rpm. *L. reuteri *biofilms were stained with acridine orange for imaging by confocal microscopy.

### Preparation of cell-free supernatants from *L. reuteri *planktonic cultures for immunomodulation studies

For planktonic cells, 10 mL of LDMIIIG was inoculated with *L. reuteri *cultures (incubated 16–18 hrs) and adjusted to OD_600 _= 0.1. Bacteria were incubated for 24 hours at 35°C in anaerobic conditions. Cells were pelleted (4000 × g, RT, 10 minutes) and discarded. Supernatants were filter-sterilized (0.22 μm pore size). Aliquots were vacuum-dried and resuspended to the original volume using RPMI.

### Preparation of cell-free supernatants from *L. reuteri *biofilms for immunomodulation studies

For biofilms grown in 24-well plates, *L. reuteri *cultures (16–18 hrs of incubation) were diluted 1:100 in 1 mL of MRS broth. Plates were incubated anaerobically for 24 hours at 35°C. Supernatants and planktonic cells were removed by aspiration, and biofilms were washed with 50 mM sodium phosphate buffer (37°C, 100 rpm, 10 minutes). One mL of LDMIIIG was added to each well, and the plates were incubated for 2 hours at 35°C in anaerobic conditions. The supernatants were filter-sterilized (0.22 μm pore size), vacuum-dried and resuspended in RPMI to the starting volume.

*L. reuteri *biofilms were cultured in flow cells supplied with MRS media for the first 23 hours followed by immersion in LDMIIIG at a flow rate of 2 mL per min in ambient atmosphere at 37°C. Biofilm supernatants were collected by sampling effluents, downstream from the chambers containing the biofilms, at the flow cell's luer lock connection after 24 hours of culture. The supernatants were filter-sterilized (0.22 μm pore size), vacuum dried, resuspended to 1/20 the starting volume in RPMI, and tested for TNF inhibition.

### TNF inhibition experiments

As previously described [45], cell-free supernatants of *L. reuteri *planktonic cell or biofilm cultures (5% v/v) and *E. coli *O127:B8 LPS (100 ng/mL) were added to human THP-1 cells (approximately 5 × 10^4 ^cells). Plates were incubated at 37°C and 5% CO_2 _for 3.5 hours. THP-1 cells were pelleted (1500 × g, 5 minutes, 4°C), and TNF quantities in monocytoid cell supernatants were determined by quantitative ELISAs (R&D Systems, Minneapolis, MN).

### Preparation of cell-free supernatants from *L. reuteri *planktonic cultures for reuterin quantification

Based on a previously described method [46], *L. reuteri *cultures (16–18 hrs of incubation) were washed twice with sodium phosphate buffer (50 mM Na_2_HPO_4 _and NaH_2_PO_4_). The cells were then suspended in sterile 250 mM glycerol at 2.2 × 10^9 ^CFU/mL and incubated anaerobically at 35°C for 2 hours. Supernatants were filter-sterilized (0.22 μm pore size) and stored at 4°C before the concentration of reuterin was determined. The quantities of reuterin were determined using a colorimetric assay previously described [46]. Briefly, serial dilutions of reuterin were made in sterile glycerol (250 mM). Forty μL of each reuterin dilution were combined with 30 μL of tryptophan (10 mM) in HCl (50 mM), and 120 μL of HCl (12 M). Under acidic conditions, tryptophan reacts with the aldehyde of reuterin to form a β-carboline derivative that oxidizes to yield a purple pigment. Plates were incubated for 25 minutes at 37°C in ambient atmosphere, and the OD560was determined using the Spectramax 340 PC384 (Molecular Devices, Sunnyvale, CA). Dilutions of HPLC-quantified reuterin were used as standards. The amount of reuterin produced was normalized to the initial CFU/mL of the cultures.

### Preparation of cell-free supernatants from *L. reuteri *biofilms for reuterin quantification

For biofilms grown in 12-well plates, *L. reuteri *cultures (16–18 hrs of incubation) were diluted 1:100 in 2 mL of MRS broth. Plates were incubated anaerobically for 24 hours at 35°C. Supernatants and planktonic cells were removed by aspiration, and biofilms were washed with 50 mM sodium phosphate buffer (37°C, 100 rpm, 10 minutes). The wash buffer was aspirated, and 2 mL of sterile 250 mM glycerol were added. The plates were incubated anaerobically at 35°C for 2 hours. Supernatants were filter-sterilized (0.22 μm pore size) and stored at 4°C before the concentration of reuterin was determined. Reuterin was produced and measured by methods described in the previous section (adapted from [29]). Biofilms were removed from multiwell plates by sonication (5 minutes, 20°C), and serial dilutions were plated to determine cell counts. The quantities of reuterin were normalized to the initial bacterial counts (bacterial cell numbers at the beginning of each experiment) of biofilms cultured under identical conditions.

### Statistical analyses

All experiments were performed a minimum of three times and analyzed using a single factor ANOVA test. Differences were considered statistically significant if p < 0.05. All error bars in the figures represent standard deviations.

## Authors' contributions

SEJ designed and undertook all experiments described in this manuscript. SEJ and JV drafted the manuscript. JV conceived the study, supervised the research and secured funding for this research. All authors have read and approved the final manuscript.

## Supplementary Material

Additional file 1 **Supplementary table.** The recipe for the medium, LDMIIIG. Click here for file
