# Supplementary material for: Probiotic Lactobacillus reuteri biofilms produce antimicrobial and anti-inflammatory factors
Source: BMC Microbiol. 2009 Feb 11;9:35. doi: 10.1186/1471-2180-9-35 (PMC2653509; doi:10.1186/1471-2180-9-35)
Supplement: Additional file 1 — Supplementary table. The recipe for the medium, LDMIIIG. [file 1471-2180-9-35-S1.doc]

Table 1. LDMIIG medium

| Solution Ingredient Quantity |
| --- |
| **Basal Medium** *^∞^* K_2_HPO_4_  1.5g  KH_2_PO_4_  1.5.g  Sodium acetate 15g  Sodium citrate 0.22g  Tryptophan 0.05g  Asparagine 0.20g  Vitamin-free Casamino acids 10g  Cysteine hydrochloride 0.20g  Water 787.5 mL  **Vitamin solution*** Thiamine hydrochloride 0.2 mg  p-aminobenzoic acid 0.04 mg  Calcium panothenic acid 0.4 mg  Niacin 1 mg  Pyridoxine hydrochloride 0.2 mg  Water 0.5 mL  **Biotin Solution***  Biotin 0.05 mg  Ethanol 0.05 mL  0.01 M HCl 0.5 mL  **Riboflavin solution*** Riboflavin 0.4 mg  0.02 M Acetic acid 5 mL  **Folic acid solution*** Folic acid 0.1 mg  0.001M NaOH 0.5 mL  **Nucleic acid solution*** Adenine sulfate 10 mg  Guanine hydrochloride 10 mg  Cytidine 5’-monophosphate 30 mg  1 M HCl 3 mL  **Uracil solution*** Uracil 20mg  1 M NaOH 1 mL  **Thymidine solution*** Thymidine 2 mg  Water 1 mL  **Salt solution*** MgSO_4_ x 7H_2_O 1.625 g  MnSO_4_ x H_2_O 0.143 g  FeSO_4_ x 7 H_2_O 0.130 g  Water 1 mL  **40% Glucose (w/v) solution*** 200 mL |

∞ Basal medium was autoclaved at 121° C for 35 minutes.

* Filter sterilize through a 0.2 μm filter (Millipore, Bedford, MA) before adding to the sterile basal media.
